# Difficulties in the Way of Nursing Reform

**Published:** 1907-04-20

**Authors:** 


### Nursing Administration.

### DIFFICULTIES IN THE WAY OF NURSING REFORM.

Among all the difficulties which are blocking the way against nursing reform, incomparably the most serious is that of forming the Board or Council which would be charged with the duty of regulating the nursing profession. Nine-tenths of the reluctance which exists among the heads of the training schools to touch the question arises from their conviction that to have no central authority is better than to have the wrong central authority, and that as matters are there is no security that a really representative Board, such as would command their confidence, would be appointed. Such a Board, charged with the State regulation of nursing, would have the definite day imposed upon it of inviting nurses to come under its dominion. It would have power to fix the conditions under which it would consent to admit members to what would be constituted officially as the main body of the nursing profession, and to remove from this body any member who should be proved to have transgressed the regulations it was empowered to make in the interests both of nurses and of the public. Training schools which did not fit their nurses to become members of this body with its State cachet might find it difficult to get probationers, and might be compelled to modify their curriculum to suit the requirements of the Board. All this is exactly what is most to be desired. But there is a big element of risk. The training schools which are at present lukewarm, if not actually hostile towards reform, have themselves evolved the nurse in the course of the last 50 years. Step by step they have won their way, often in the teeth of overwhelming financial difficulties, towards a training system which, whatever its defects, can boast of producing incomparably the best trained nurses in the world. Is it surprising that they are reluctant to watch the control pass out of their hands to a body elected on lines which leave them without any security for its policy?

But, say the ardent advocates of change, the training schools must be coerced. When the Board is once constituted all these imaginary terrors will melt away, and they will begin to regard it as their best friend. It may be quite true that the proposed Board may, under whatever conditions elected, prove a far more reasonable body than some people anticipate. But it is undeniably true that, should the training schools refuse to support the Board, should they agree upon united action to ignore its provisions, it might remain an empty and somewhat costly shell, futile alike for good or evil. The rather acrid discussions on registration which have gone on for so long have cleared the ground from a great deal of foolish misapprehension on the subject. Nobody now hears anything about the compulsory registration of nurses, which used in old days to be the war-cry. Nobody talks of penalising trained nurses who may refuse to be registered, or untrained nurses who may refuse to be trained. All that has melted away in the light of anticipated legislation. That the whole system must be purely voluntary is now universally recognised. The voluntary system has been tried already and has failed. The registration system of the Royal British Nurses' Association, framed expressly with the aim of conciliating the training schools, resting solely on the recommendation of the matron and the certificates of certain recognised hospitals, has failed to command the support of the great body of trained nurses. The reason was simply that the nurses have found themselves getting on very well without it.

Should the heads of training schools unanimously advise their nurses against accepting the State guarantee of their abilities offered by a Central Nursing Board, does anyone seriously believe that these nurses would fail to get employment? The tendency would undoubtedly be for the nurses whose qualifications were in any degree inferior, let us say veering on the minimum, to accept eagerly the offered seal of efficiency, but in the best Nursing Co-operations they would find themselves still at a disadvantage with regard to nurses trained in hospitals of world-wide repute which had combined to ignore the necessity for any guarantee but their own certificate. If the nurses of the best hospitals stood out of registration the public would be in the position of having to discriminate between the nurses who considered themselves too good and the nurses who would not admit that they were too bad, for registration; while the middle sort would be wondering what advantages they were getting for their guineas.

The movement in favour of the registration of teachers has been in progress for many years, and was grounded on the existence of evils with regard to incompetent teachers at least as far-reaching as the abuses complained of in the nursing world. In the result, about five years ago the register was opened under the direction of the Board of Education, and regulations were adopted for admitting teachers to the roll, and for recognising schools under certain conditions as training centres for teachers. But the teachers of assured qualifications, University degrees, and diplomas from the big training colleges, found no benefit from entering their names, and did not in effect do so; while the big public schools, with a few spirited and wide-minded exceptions, ignored the opportunity of being inscribed as "recognised." A short time back the register was suspended, and its ultimate future is very doubtful.

With these examples in view it will be seen that the principal, we might almost say the only, difficulty which blocks the way in nursing reform to-day is the constitution of a Board able to rely on the cordial co-operation of the large training schools without whose support legislation would be worse than useless.

